# Exogenous Players in Mitochondria-Related CNS Disorders: Viral Pathogens and Unbalanced Microbiota in the Gut-Brain Axis

**DOI:** 10.3390/biom13010169

**Published:** 2023-01-13

**Authors:** Irene Righetto, Matteo Gasparotto, Laura Casalino, Marcella Vacca, Francesco Filippini

**Affiliations:** 1 Synthetic Biology and Biotechnology Unit, Department of Biology, University of Padua, via Ugo Bassi, 58/B, 35131 Padua, Italy; 2 Institute of Genetics and Biophysics “A. Buzzati Traverso”, CNR, via Pietro Castellino, 111, 80131 Naples, Italy

**Keywords:** neurological disorder, neurotropic virus, Influenza A, SARS-CoV-2, coronavirus, COVID-19, Long COVID, gut microbiota, Spike, Neuropilin, hemagglutinin, cognitive function

## Abstract

Billions of years of co-evolution has made mitochondria central to the eukaryotic cell and organism life playing the role of cellular power plants, as indeed they are involved in most, if not all, important regulatory pathways. Neurological disorders depending on impaired mitochondrial function or homeostasis can be caused by the misregulation of “endogenous players”, such as nuclear or cytoplasmic regulators, which have been treated elsewhere. In this review, we focus on how exogenous agents, i.e., viral pathogens, or unbalanced microbiota in the gut-brain axis can also endanger mitochondrial dynamics in the central nervous system (CNS). Neurotropic viruses such as Herpes, Rabies, West-Nile, and Polioviruses seem to hijack neuronal transport networks, commandeering the proteins that mitochondria typically use to move along neurites. However, several neurological complications are also associated to infections by pandemic viruses, such as Influenza A virus and SARS-CoV-2 coronavirus, representing a relevant risk associated to seasonal flu, coronavirus disease-19 (COVID-19) and “Long-COVID”. Emerging evidence is depicting the gut microbiota as a source of signals, transmitted via sensory neurons innervating the gut, able to influence brain structure and function, including cognitive functions. Therefore, the direct connection between intestinal microbiota and mitochondrial functions might concur with the onset, progression, and severity of CNS diseases.

## 1. Introduction

The central nervous system (CNS) is known to be the most energy demanding system in the human body. It is estimated that a single neuron requires approximately 4.7 billion ATP molecules per second, even in its resting state [1]. Such a huge amount of energy is required for the homeostasis of the membrane potential and for the release of neurotransmitters (and their recapture from the synaptic cleft) [1]. Therefore, mitochondria are crucial for CNS functionality. However, despite having been considered for decades as just “power plants” in eukaryotic cells, recent research on mitochondria proved these organelles are involved in a much larger number of important regulatory processes at cellular, tissue, organ, and organism level, which - when impaired - can determine a wide plethora of diseases.

In the first special issue of a series focusing on mitochondria-related disorders of the CNS, we focused on “endogenous players”, i.e., on events occurring from chromatin remodeling and transcriptional control to post-transcriptional events, vesicle, and organelle biogenesis, fusion, and subcellular trafficking, which - when impaired - can alter mitochondrial function and determine neurodevelopmental or neurodegenerative diseases [2]

**Figure 1:**
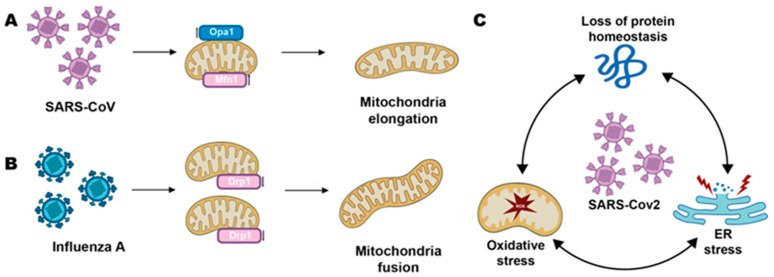
Viruses can impair fusion and fission of neuronal mitochondria via direct interaction with their proteins (**A**,**B**) or indirectly, via oxidative/ER stress (**C**). Examples are reported in Section 2, Section 3 and Section 4.

In this review, instead, we will illustrate how impaired mitochondrial function, resulting in CNS diseases, may depend on “exogenous players” like viral pathogens and an unbalancing of the microbiota in the gut-brain axis. All abbreviations used are listed in Abbreviation section. Indeed, mitochondria can both be hijacked by pathogens for intracellular replication and spreading by direct interaction with mitochondrial proteins, and thus alter the fusion or fission of these organelles and be indirectly damaged via oxidative/endoplasmic reticulum (ER) stress (Figure 1).

Although mitochondria have been reported to play an antiviral role during viral infection, several viruses can hijack them to infect different cells [3], including neurons. Besides neurotropic viruses (such as herpes, rabies, West-Nile, and Polioviruses) other viruses can occasionally infect CNS neurons, although they have different tissues/organs as primary targets. 

An example of a CNS disease caused by a non-neurotropic virus is the human immunodeficiency virus (HIV)-associated neurocognitive disorder (HAND), which comprises of the impairment of multiple cognitive domains and is a leading cause of morbidity in up to 50% of individuals living with HIV [4,5]. Among HIV proteins involved in HAND-associated neurodegeneration, Tat (trans-activator of transcription) was found to induce a [Ca2+] increase in cultured embryonic rat hippocampal neurons, with an effect on mitochondria, mitochondrial reactive oxygen species (mtROS) accumulation, and induction of neuronal apoptosis. Calcium increase is also mediated by HIV glycoproteins gp120 and gp160 [6].

Another non-neurotropic virus that can cause mitochondria-related CNS damage is Human T-cell leukemia virus type 1 (HTLV-1), which is a retrovirus that, like HIV, has another primary cell target and this, notwithstanding, can infect the brain and lead to spastic paraparesis. HTLV-1 accessory protein p13II is responsible for changes in Ca^2+^ uptake and retention, acting on mitochondrial permeability alteration, membrane potential-dependent influx of K^+^ generation, mitochondrial matrix volume increasing, and fragmentation [7].

Patients infected by pandemic viruses such as Influenza A virus and SARS-CoV-2 coronavirus can also develop CNS disorders, of which some are seemingly mitochondria-related, and they will be treated in next specific sections.

Among external cues, the gut microbiota is the source of a number of bioactive metabolites [8] which shape the structure and function of brain regions involved in the control of emotions, cognition, and physical activity [9,10]. Bacteria may signal to the brain through multiple routes, including neural, metabolic, endocrine, and immune pathways. In addition to direct sensing of bacteria by sensory neurons innervating the gut, a metabolic communication with the brain occurs when bacterial metabolites become absorbed into the portal vein, enter circulation, and may then reach CNS by crossing the blood-brain barrier (BBB). Bacterial metabolites provide energy for eukaryotic cell metabolism [11], and neuro and inflammatory modulators, but also mitochondrial toxins. In the second part of this review, we will describe the so-called gut-brain axis (GBA) and discuss how the direct connection between intestinal microbiota and mitochondrial functions might concur with the onset, progression, and severity of CNS diseases.

## 2. Neurotropic Viruses

Mitochondria can prevent the abnormal increase in cytoplasmic calcium concentration; therefore, impaired mitochondrial transport into the presynaptic terminal is responsible for neurotransmission dysregulation [12]. Neurotropic viruses can alter calcium intracellular levels, as [Ca2+] activates fundamental pathways required for viral replication and provides a persistent infection [13]. The ER is a target for the virus, due to its intracellular calcium stores. Acting on ER channels, viruses make it possible for the release of a higher amount of Ca^2+^ in the cytoplasm, which is then used by mitochondria to boost ATP production to provide the higher energy cellular demands required for continuous viral replication. Moreover, a decrease of calcium concentration in the Golgi apparatus and ER fights the anti-viral response and the regulation of ER-[Ca2+] crosstalk may facilitate or prevent apoptosis [13]. Viral infections of the brain often cause death of neurons and astrocytes. Some neurotropic viruses, such as CHPV (Chandipura Virus) and JEV (Japanese Encephalitis Virus), share common host proteins modulating pathogenesis, as revealed by network analysis [14]. In this case, the DJ-1 protein is over-expressed in response to ROS generation and is able to modulate the viral replication and interferon responses along with low-density lipoprotein (LDL) receptor expression in neurons.

### 2.1. Poliovirus

Poliovirus (PV) is responsible for the destruction of motor neurons via apoptosis, leading to paralytic poliomyelitis. PV is able to induce a [Ca2+] _cyt_ increase. This event takes place via inositol-1,4,5-triphosphate receptor (IP3R) and Ryanodine receptor (RyR) channels [15], resulting in a drop in membrane potential. Calcium from the ER is accumulated in the mitochondria through voltage-dependent anion channel-1 (VDAC), which is located on the outer mitochondrial membrane (OMM), and the mitochondrial calcium uniporter (MCU) that is located on the inner mitochondrial membrane (IMM). This results in mitochondrial dysfunction, apoptosis, and improved virus spreading. On the contrary, the non-structural protein 2B is responsible for the Ca^2+^ increase from extracellular sources, decreasing at the same time the [Ca2+] _ER_ and [Ca2+] _Golgi_. This way, the host cell apoptosis is suppressed via the inhibition of caspase 3 activation, ensuring virus replication [16].

### 2.2. Herpes Virus

Herpes simplex virus (HSV) was found to increase [Ca2+] in neurons due to increased firing. This affects the interaction of mitochondrial protein Miro-1 with kinesin-1, resulting in the disruption of mitochondrial mobility and allowing viral spread. ND7/23 sensory neurons infected by HSV exhibit a modulation of [Ca2+] by T-type voltage gated calcium channels (VGCC) [17].

HSV-1 encephalitis (HSE) is the most common cause of viral encephalitis and a severe disease with high morbidity and mortality. Post-mortem human HSE brains show a high reduction in mitochondrial transcripts compared to controls, demonstrating that mitochondrial damage underlies the HSE phenotype, and this is confirmed in a primary human astrocyte HSV-1 infection model [18]. It is noteworthy that the same decrease in the expression of many genes important for mitochondrial function, observed with HSV infection, can also result in oxidative stress and neuronal damage leading to Alzheimer’s disease [19].

### 2.3. Rabies Virus

The Rabies virus (RABV) belongs to the lyssavirus genus of the Rhabdoviridae family and is the etiological agent of rabies with severe neurological symptoms and 100% mortality. RABV is able to induce apoptosis in vitro and in vivo. This virus carries a single non-segmented negative strand RNA genome. Five genes are encoded: the nucleoprotein (N), phosphoprotein (M), matrix protein (M), glycoprotein (G), and the viral RNA polymerase (L). Protein M plays a role in apoptosis induction as it partially targets mitochondria activating caspase-dependent and caspase-independent pathways at the late stage of infection. In particular, caspase-9 and caspase-3 are activated in a time-dependent manner, but not caspase-8. This phenomenon implies that the apoptosis induced by RABV involves the mitochondrial intrinsic pathway. At the late stage of infection, mitochondrial membrane potential is significantly dissipated, and the cytochrome c diffuses into the cytoplasm from mitochondria. Moreover, infection by RABV elicits the expression of the proapoptotic protein AIF, translocating into the nucleus of the infected cells [20]. RABV infection also leads to m-calpain upregulation, providing a proof of altered [Ca2+] _m_ uptake. M-calpain cleaves Bid, activates calcineurin A, and stimulates killer kinases, thus leading to the cell death [21].

### 2.4. West-Nile Virus

West Nile virus (WNV) is a single stranded RNA flavivirus and a member of the JEV serocomplex. Even though WNV infections in humans most often result in mild illness, 0.5–1% of the patients may develop meningitis, encephalitis, acute flaccid paralysis, and death due to neurotropic invasion of the CNS and neuronal apoptosis [22]. Apoptosis is accompanied by the release of cytochrome c from the mitochondria and the formation of apoptosomes [23]. Neuro-2a cells (neuronal cells) show typical apoptotic characteristics when infected by WNV, e.g., Bax gene up-regulation [24]. Brain-derived T98G cells can undergo both extrinsic and intrinsic apoptotic pathways upon WNV infection, and Caspase-3-dependent neuronal death contributes to the pathogenesis of West Nile virus encephalitis [25,26].

### 2.5. Zika Virus

Zika virus infection can result in microcephaly in newborns, but serious neurological complications can also concern adults. Indeed, Zika virus is able to cause cell death in neural progenitor cells, astrocytes, and neurons. Studies in mice embryo brains showed that primary neurons in the cortico-striatal region exhibit an increased expression of the N-methyl-D-aspartate receptor (NMDAR) subunit GluN2B and a [Ca2+] increase [27]. Excess [Ca2+] _ER_ released from the lumen is then taken up by the mitochondria, leading to an increase in mtROS and the subsequent DNA damage [28].

The NS4B protein of Zika virus has been reported to recruit Bax to the mitochondria and induce Bax conformational activation. The overexpressed NS4B was localized to the mitochondria and induced cell apoptosis by activating the pro-apoptotic protein Bax [29]. Since astrocytes are targets of the Zika virus, iPSC-derived astrocytes were used as a model system to investigate key mechanisms and fates involved in the neurotoxicity of the virus, finding that Zika virus infection leads to mitochondrial failure, oxidative stress, and DNA damage [30].

## 3. SARS-CoV-2

RNA viruses are commonly responsible for several known and emerging infections. These viruses spread from animal reservoirs and are able to adapt to new hosts [31]. Among RNA viruses, coronaviruses (CoVs) can be divided into four genera: Alphacoronavirus, Betacoronavirus, Gammacoronavirus, and Deltacoronavirus, of which the first two only infect mammals (including human CoV) [32]. Human CoVs cause respiratory infections that can result in quite varying morbidity, from very mild or asymptomatic common cold, to severe pneumonia, acute respiratory distress syndrome (ARDS), and even death [33]. Roughly twenty years ago, an outbreak of Severe Acute Respiratory Syndrome coronavirus (SARS-CoV), followed by a geographically more limited outbreak of Middle East Respiratory Syndrome (MERS) coronavirus, caused an aggregate death toll of a few thousands patients worldwide [34], while, unfortunately, SARS-CoV-2, the causative agent of the acute coronavirus disease 2019 (COVID-19), has become a pandemic since 2020, and has caused so far millions of deaths and more than half a billion infections (https://www.worldometers.info/coronavirus/; accessed on 2 November 2022).

SARS-CoV-2 infection is able to disorganize the immune response, impairing and delaying type I interferon (IFN-I) response, and sometimes eliciting a “storm” of proinflammatory cytokines, which in turn brings serious illness and eventually death [35].

Targeting of the IFN system is mediated by the interaction of CoV accessory proteins with mitochondria anti-viral signaling (MAVS) adaptor molecules, e.g., SARS-CoV ORF3b targets MAVS to inhibit IFN induction [36]. ORF9b from both SARS-CoV and SARS-CoV-2 target MAVS to reduce IFN production, showing a conserved mechanism [37,38,39]. SARS-CoV ORF3a protein can activate caspases 8 and 9, determining cytochrome c release from the mitochondria and modulating the mitochondrial death pathway [40]. SARS-CoV-2 non-structural proteins (Nsp), e.g., Nsp2 and Nsp4, are also reported to interact with mitochondria [41], as well as Nsp13 [3]. Both SARS-CoV N and M proteins induce the release of cytochrome c, mediating apoptosis [42,43]. In particular, the SARS-CoV-2 M protein is able to impair viral RNA-induced MAVS through downstream components [44].

In addition to direct interaction with mitochondria, SARS-CoV-2 is able to impair the homeostasis of these organelles by altering, via gastrointestinal infection, the crosstalk between mitochondria and the gut microbiota [45]. Signaling in such crosstalk occurs by means of endocrine, immune, and humoral links [46], that, when impaired, determine the unbalancing of the gut-brain axis and subsequent CNS damage.

Apart from millions of deaths and morbidities mainly mediated by the well-known damage to the respiratory system, COVID-19 created in infected people major neurologic complications, which often persist after recovery [47]. SARS-CoV-2 presence in the CNS, and especially in the brain, is confirmed by autopsies of patients [48]. COVID-19 patients may exhibit a wide plethora of neurologic symptoms, from mild such as headache, confusion, and nausea, to more severe, like loss of consciousness, anosmia and ageusia (loss of sense of smell and taste), Guillain–Barre syndrome (GBS), cerebral inflammation, meningitis and encephalitis, strokes, and seizures [49].

Entry of SARS-CoV viruses into host cells occurs via clathrin-mediated endocytosis (CME), and neuronal CME is similar to non-neuronal CME, although neuron-specific isoforms of the adaptor complex proteins mediate synaptic endocytosis [50]. In addition to major players such as the functional receptor angiotensin converting enzyme 2 (ACE2) and transmembrane serine protease 2 (TMPRSS2), SARS-CoV-2 spreading in the CNS is further favored by the binding of the coronavirus Spike protein to Neuropilin-1 [51], which is transported into the cell following the retrograde pathway of the endosomal SNX-BAR sorting complex promoting exit 1 [52].

Molecular docking simulations have suggested that binding to Neuropilin-1 of the Spike protein by the most recent Omicron variant of SARS-CoV-2 is energetically more favorable, at least in part providing a rationale for the higher infectivity of this variant [53]. In normal conditions, Neuropilin-1 is involved in the angiogenesis and development of neuronal circuits [54]; moreover, this protein is able to modulate the cytokine profile by influencing the Fas receptor-Fas ligand signaling, which in turn is involved in the virus’ entry into the cell [55]. When considering that the cytokine storm is a major determinant in a severe outcome of COVID-19, it is noteworthy that Neuropilin-1 is both a functional receptor for SARS-CoV-2 and a modulator of the cytokine profile, suggesting a molecular rationale for neurological complications and a target for therapeutics [55]. Neuropilin-1 also plays a pivotal role in the homeostasis of endothelial cells (ECs), promoting mitochondrial function in ECs by interacting with the mitochondrial transporter ATP-binding cassette B8 to prevent iron accumulation and iron-induced oxidative stress [56]. Neuropilin-1 mediated infection and damage of ECs also occurs at the BBB, representing the CNS entry route for SARS-CoV-2 and subsequent neurological complications [57].

SARS-CoV-2 infection may result in long-term effects on immune processes within the CNS by causing microglial dysfunction and after COVID-19 post-acute multi-organ complications have been widely reported, altogether referred to as “Long COVID”. Long COVID also includes the so-called “brain fog”, consisting of serious and long-term impairment of mental and cognitive performance. It has been proposed that, upon SARS-CoV-2 infection, selective mitochondrial targeting in CNS neurons affects cognitive processes to induce ‘brain fog’ associated behavior that favors viral propagation in the population [58].

## 4. Influenza A Virus

Influenza A viruses infect not only the respiratory system, but also the CNS. These viruses are able to infect avian and mammalian hosts, using their high mutation rate [59] as a strategy to evade the immune response and/or perform a host jump or pathogenicity shift. Indeed, mutations mediating changes in surface features such as hydropathy and electrostatics of the two major antigens, hemagglutinin (HA) and neuraminidase (NA), modulate the virus’ affinity to different hosts and cell types [60,61,62].

Encephalopathy, encephalitis, Reye’s syndrome, acute necrotizing encephalopathy, and myelitis, as well as autoimmune conditions such as GBS, are influenza-associated disorders [63,64]. Entry of these viruses into the CNS can occur via the olfactory, trigeminal, vagus, and sympathetic nerves, and possibly other cranial nerves [65]. Despite the presence of the BBB, Influenza (and other) viruses can invade the brain. This can be achieved via different mechanisms (e.g., paracellular, transcellular, and/or by a “Trojan horse” pathway) [66]. Influenza A virus infection of astrocytes can impair neural turnover of mitochondria as these cells are able to take up and degrade damaged mitochondria secreted by neighboring neurons via trans-mitophagy [67], as well as to secrete functional mitochondria for neurons to survive in harmful situations such as a stroke [68]. HA, NA, matrix (M), and non-structural (NS) genes play an important role in determining neurovirulence [69].

A neuroinvasive H5N1 Influenza virus emerged in Hong Kong in 1997 and HA was found to play a central role in high lethality [70,71]. Severe human cases of zoonotic infection by avian Influenza virus are often accompanied by neurological symptoms, and it has been found that adaptive HA mutations allow the virus to infect mouse and human brains via avian-like sialic acid receptors [72]. In hemagglutinin, mutations at its multi-basic cleavage site (MBCS) [65] and/or at specific loops [73] have been associated with the low-pathogenicity (LPAI) to high-pathogenicity (HPAI) shift. Indeed, mutations at the MBCS also modulate the Influenza virus capacity to infect neuroblastoma-derived SK-N-SH cells and U87-MG glial cells [65].

Mutations in the NA gene can determine enhanced pathogenicity and neurotropism in a mouse adapted to the H10N7 Influenza virus [74]. Changes in the NS gene cause alterations in the mRNA secondary structure to mask the 3′ splice site and correlate with reduced splicing of the NS gene in neurovirulent strains [69].

Once neuronal cells are infected, a number of other Influenza A virus proteins can counteract the hosts innate immunity and interact with mitochondria. Influenza A matrix protein M2 is a homo-tetrameric integral membrane protein, showing proton channel activity [75]. This protein is crucial in viral entry and synthesis of new viruses during viral particle assembly and budding. M2 colocalizes and interacts with MAVS proteins in mitochondria, inducing a positively regulated MAVS-mediated innate immunity. This viral protein is able to anchor to mitochondria, promoting mitochondria fusion and increasing their number. Influenza A virus infection modifies mitochondria morpho-dynamics, promoting mitochondria hyper elongation by fission associated protein DRP1 re-localization to the cytosol, enhancing a pro-fusion status [76]. Moreover, M2 protein is involved in the production of ROS due to its proton channel activity, required for the activation of macroautophagy/autophagy and enhancement of MAVS signaling pathway. M2 is able to trigger extracellular Ca^2+^ influx-dependent ROS production, subsequently leading to the activation of autophagy related 5 (ATG5) protein and the inhibition of AKT serine/threonine kinase and of the mechanistic target of rapamycin (MTOR) kinase activity via class I phosphoinositide 3-kinase (PI3K)-AKT-MTOR signaling pathway, which then activates autophagy [75].

Another viral protein, named PB1, is involved in the inhibition of the hosts innate immune response [77]. PB1 is an essential factor of the Influenza A virus RNA polymerase complex for viral transcription and replication. It acts as a negative regulator of virus—or poly (I:C)—stimulating interferon induction and interacts with MAVS, destabilizing it. This viral protein is able to enhance the degradation of MAVS by promoting a signaling cascade. The pathway starts from the E3 ligase RNF5-mediated ubiquitination of MAVS, then follows the recruiting of the selective autophagic receptor NBR1 to associate with, and finally the delivering of the ubiquitinated MAVS to the autophagosomes for degradation takes place. This way, the MAVS-mediated innate signaling pathway is abolished, leading to the suppression of IFN-I induction and facilitation of virus replication.

In addition to PB1, PB2 can also interact with mitochondria. More specifically, PB2 is imported into the mitochondrial matrix [78]. Like PB1, PB2 is able to inhibit IFN expression by associating with MAVS, which acts downstream of retinoic acid-inducible gene-I (RIG-I) and cellular RNA receptor MDA-5 in the IFN induction pathway. Its import into the mitochondrial matrix is ensured by residue 9 of PB2, which typically is asparagine or threonine. Intriguingly, the RIG-I signaling pathway has been found to contain the targets of COVID-19-related long non-coding RNAs [79]. 

Mitochondrial damage after Influenza A pulmonary infection can lead to adverse cardiac events as viral particles can persist in the heart after lung clearance, altering mitochondrial function and promoting cell death without active replication and IFN responses [80]. Influenza infection in cardiomyocytes showed changes to mitochondrial function, increased oxidative stress, and cellular toxicity. Apoptosis can be achieved also via Bcl-2 antagonist of cell death (BAD)-mediated mitochondrial dysregulation [81]. BAD is a cell death regulator, crucial in the intrinsic pathway of apoptosis, occurring thanks to the dysregulation of the OMM permeabilization due to the change of mitochondrial membrane potential [82] and the subsequent release of apoptogenic factors. In this scenario, the viral NS1 protein interacts with Akt, which results in enhanced Akt activity. BAD S136 phosphorylation takes place via PI3K signaling pathway.

## 5. Unbalancing of the Gut-Brain Axis

**Figure 2:**
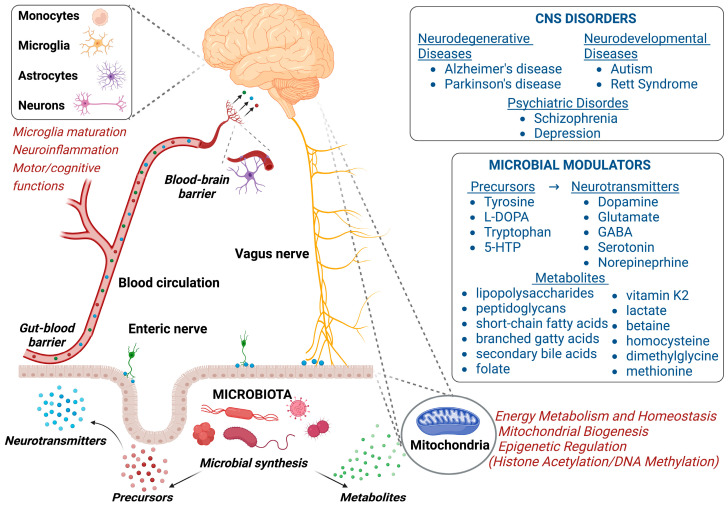
Schematic model of the gut microbiota-brain axis interplay. The crosstalk between gut microbiota and mitochondria is involved in many CNS disorders, as reported in the right-upper panel. Microbial modulators are listed in right-lower panel. Left figure: Neurotransmitters and their precursors, as well as metabolites, produced directly by the gut microbiota and/or through dietary metabolism can cross the gut-blood barrier and enter the bloodstream. Some of them can cross the blood–brain barrier to directly affect brain functions (microglia maturation, neuroinflammation, and motor and cognitive functions). In addition, gut microbiota-modulated changes in neurotransmitter/precursor synthesis may also lead to alterations in sensory signals transmitted to the brain through the enteric and vagus nerves. Finally, the microbiota metabolites also affect the hosts energy metabolism, homeostasis, and gene expression by influencing the in situ mitochondria of the gut epithelium and distal mitochondria of the brain cells. Created with BioRender.com (accessed on 2 November 2022).

The CNS and the human gastrointestinal (GI) tract communicate through the enteric nervous system and the gut-brain axis (GBA). Such communication of the GBA is bi-directional and involves neuronal, endocrine, and immunological mechanisms, and also includes the peripheral nervous system. The interplay between GBA and intestinal microbiota may play an important role in brain development and homeostasis. Neurons are particularly enriched in mitochondria to satisfy their huge energy demand. Consequently, the maintenance of mitochondria homeostasis is fundamental to physiological differentiation, connectivity, and functionality of neural cells (Figure 2). Recently, an interesting evolutionary perspective has pointed to the possibility that mitochondria could be at the crossroad between brain and gut microbiota, thus favoring their bi-directional communications [83].

The microbiota in mammals is a highly enriched community of prokaryotes, fungi, and viruses that participate in their physiology [84]. Microbiota is spread over different barrier surfaces and anatomical niches, but here we will focus on the gut and resident bacteria. The bacterial community of gut microbiota includes symbionts, commensals, and pathogens, and its composition is highly dynamic and sensitive to lifecycle, environmental, immune, and diet. Accordingly, different bacterial products could in turn either modulate host physiology (metabolism, immune system, and gene expression) or induce pathogenic effects [85,86]. The imbalance in the gut microbial community, due to the gain or loss of community members or changes in the relative abundance of microbes, is defined as dysbiosis. Disruption to the microbiota might be associated with conditions, stress—physical and psychological—or results from antibiotic therapies. Pediatrics and progressive neurodevelopmental disorders (NDDs) are related to dysbiosis, including an increased load of pathogens and opportunistic microbes. Particularly, antibiotics administered throughout the first 18 months of life might influence the development of a child’s nervous system and contribute to NDDs by creating microbiota imbalances [87].

The gut microbiota can also influence brain function and behavior by signaling through the immune system, both peripherally and in the CNS. For instance, the gut microbiota affects circulating levels of pro- and anti-inflammatory cytokines, which can act on corresponding receptors in the brain endothelium, microglia, astrocytes, and neurons. Bacterial metabolites also strongly influence the innate immune functionality of microglia, the resident myeloid immune cells in the brain, by modulating its maturation and activation. Microglia play critical roles in synapse pruning, clearance of apoptotic bodies, and signaling to neurons in the brain in health and neurological diseases. Of note, chronic neuroinflammation has been documented in many progressive brain disorders either with multifactorial (e.g., Alzheimer Disease [88], Parkinson disease [89], and autism spectrum disorders (ASD) [90]) or monogenic origin (e.g., Rett syndrome (RTT) [91]). 

### 5.1. Gut Bacterial Modulation of Mitochondrial Homeostatic Function

Strikingly, neuroinflammation is also associated with oxidative stress and mitochondria dysfunction, particularly through ROS production, highlighting the complex convergence of molecular mechanisms and organelles involved. On the other hand, changes in mitochondrial redox homeostasis have been shown to modulate intestinal barrier function and mucosal immune responses, and thus contribute to regulating the gut microbiota. Furthermore, genetic variants within the mitochondrial genome could affect mitochondrial function and, in turn, the intestinal microbiota composition and activity.

Current high-throughput sequencing technologies and metabolomic approaches are providing new important information about the composition and functionality of the gut microbiome [92]. Of note, recent evidence revealed that the bi-directional interaction between neural mitochondria and microbiota overcomes oxidative stress.

For example, the gut microbiota has been shown to regulate key transcriptional co-activators, transcription factors, and enzymes involved in mitochondrial biogenesis, such as PGC-1α, sirtuin 1 (SIRT1), and AMPK; other metabolites might influence energy production and epigenetic signatures [93,94]. Bacteria-derived molecules encompass lipopolysaccharides, peptidoglycans, short-chain fatty acids (SCFA), secondary bile acids [95,96], folate [97], vitamin K2 [98], lactate [99], neurotransmitters such as choline, dopamine, noradrenaline, serotonin, and GABA [85], and gaseous molecules [100].

SCFAs, including acetate, propionate (PPA), and butyrate, result from the fermentation of non-digestible dietary fibers, carbohydrates, and some proteins, and represent powerful mitochondrial regulators. Following absorption into the circulation [101,102], SCFAs affect the hosts energy metabolism [103] and homeostasis by acting on G-protein coupled receptors (GPCRs). Apart from being used as bacterial energy substrates in the intestine, SCFAs regulate fatty acid and glucose metabolism through an AMPK-PGC-1α -dependent mechanism and are also capable of increasing mitochondrial bioenergetics and biogenesis [104,105]. SCFAs are also reported to regulate inflammation, by stimulating the maturation of microglia, inhibiting autophagy and TNFα-mediated immune responses, and targeting inflammasomes such as NLRP3 [106]. Among SCFAs produced by the gut, microbiota butyrate acts as a potent histone deacetylase (HDAC) inhibitor. Interestingly, acetylated proteins of mitochondria which participate in oxidative phosphorylation and tricarboxylic acid cycle have recently been proposed as key molecules by which gut microbiota might regulate brain function and behavioral phenotypes [107].

Bacterial methyl metabolites, including betaine, homocysteine, dimethylglycine, and methionine, link host lipid accumulation with environmental methionine availability. Prokaryotic supply of lactate can concur to SIRT1 deacetylase upregulation in the hippocampus, leading to brain-derived neurotrophic factor (BDNF) expression [108] that modulates neural plasticity. Moreover, SIRT1 is a sensor of the cytosolic ratio of NAD(+)/NADH which is altered by glucose deprivation and metabolic changes associated with caloric restriction [109].

Microbiota metabolites thus provide a mechanism of epigenetic crosstalk between the nucleus and mitochondria. Accordingly, microbiota can regulate the methylation of nuclear and mitochondrial DNA and the transcriptome of the host. Germ-free mice enabled the dissection of microbiota-dependent and independent sex- and time-dependent processes during postnatal development acting to shape DNA methylation and transcriptome signatures of intestinal epithelial cells [110] and microglia. In particular, differentiation phases of microglia correspond to microbiota-dependent transcriptomic signatures and chromatin accessibility landscapes, which can diverge in adult males and females. Similarly, SIRT1 can modulate chromatin function through deacetylation of histones and can promote alterations in the methylation of histones and DNA, leading to transcriptional regulation [111].

### 5.2. Microbiota and GBA Interplay in Mitochondria-Related CNS Disorders

Gut dysbiosis may influence the severity of brain disorders with unpredictable consequences on phenotypic heterogeneity in patients carrying roughly the same genetic background. Specific bacterial signatures were found in many conditions, leading to the hypothesis that microbiota changes could trigger or exacerbate neurological features.

Indeed, gastrointestinal comorbidities have been found in many neurological diseases, as exemplified by ASD [112], as well as by chromatinopathies like RTT [113] and Rubinstein-Taybi syndrome [114]. Interestingly, those conditions have been also associated with mitochondrial dysfunction [2,115].

Patients with ASD show specific metabolic features, such as carnitine deficiency and altered lactate/pyruvate ratios [116]. Carnitine enables the transport of fatty acids into mitochondria for β-oxidation and energy production [117]. In fecal samples from patients with ASDs PPA is overrepresented and, accordingly, PPA treated rats show many ASD-related symptoms, such as neuroinflammation, increased oxidative stress, intracellular acidification, and altered brain phospholipid/acylcarnitine profiles, contributing to mitochondrial impairments [116]. 

RTT is caused by mutations in the methyl CpG binding protein 2 (MECP2) gene, codifying a reader of DNA methylation. RTT is the most prevalent monogenic neurodevelopmental disorder in females and mitochondrial/metabolic dysfunctions, as well as neuroinflammation have been reported in affected patients [118,119]. Accordingly, two studies have demonstrated that patients with RTT have an altered gut microbial community compared to healthy controls [120,121]. In addition, in a representative female mouse model of RTT, mutants had higher fecal levels of butyrate, iso-valerate, and propionate than wild-type females. Genotype-related differences are not shared by age-matched male mutant mice and occur prior to the onset of neuromotor phenotypes, suggesting that gut dysbiosis might be the earliest phenotypic manifestation of RTT and play a role in disease pathology [91].

The Rubinstein-Taybi syndromes (RSTS1, MIM #180849, and RSTS2 MIM #613684) are severe neurodevelopmental disorders caused by pathogenic variants in the genes encoding the histone acetyltransferases CBP and p300, respectively. Pharmacological therapy with HDAC inhibitors was shown to attenuate chromatin impairment, improving the clinical phenotype. By analyzing the overall composition of bacterial metabolites from commensal gut microbiota, RSTS patients compared to healthy siblings showed significant depletion in butyrate [122]. Accordingly, ameliorative effects of butyrate, relative to other HDAC inhibitors, have been reported in both in vitro and in vivo models of RSTS. 

Growth retardation, developmental delay, coarse facies, and early death (GDFD, MIM #612938) is an autosomal recessive multiple congenital syndrome characterized by severe psychomotor retardation, poor overall growth, and dysmorphic facial features [123]. GDFD is caused by mutations in the fat mass and obesity-associated gene (FTO) that encodes for an mRNA demethylase [124]. By comparing hypothalamic Fto levels, germ-free mice displayed lower Fto expression than conventionally raised (Conv) mice. Notably, hypothalamic Fto expression is recovered to Conv levels by the restoration of the gut microbiota in germ-free mice. Fto deficient mice harbor a specific gut bacterial signature. Critically, behavioral alterations of female mutant mice are mediated by a shift in gut microbiota, as such changes can be partially attenuated using antibiotics [125].

Mutations in Host Cell Factor C1 (HCFC1) transcription factor cause Methylmalonic aciduria and homocystinuria (MIM #309541, also known as Mental retardation, X-linked 3), an X-linked recessive metabolic disorder characterized since early infancy by severe delayed psychomotor development. HCFC1 pathogenic variants are linked to inborn errors of cobalamin (vitamin B12) metabolism [126]. Defective HCFC1 affects the expression of MMACHC, the intracellular cobalamin transporter, thus impairing the reduction of cobalamin into the two bioactive forms that function as co-factors for both cytosolic methionine synthesis and mitochondrial β-oxidation of dietary odd-chain fatty acids [127].

## 6. Conclusions

After decades in which mitochondria have been considered important organelles, being essential for cellular energy metabolism, emerging evidence has progressively demonstrated they are central to many regulatory pathways in animal and human cell types, tissue, and organ districts, including of course neural cells and the CNS. 

Subsequently, when mitochondrial function(s) and homeostasis are impaired, several complications and disorders may occur, ranging from mild traits to severe morbidity and even death.

Mitochondria-related CNS disorders can depend on either endogenous or exogenous players, and the formers—specifically at the nuclear and cytoplasmic machinery levels—have already been treated in a review published in the first special issue of this series [2]. This review, instead, focuses on microbial, exogenous players, i.e., on viral pathogens that are causative for mitochondria-related CNS disorders, and on how an imbalanced microbiota can result in disorders as well because of altered signaling along the gut-brain axis.

Mitochondria are known to play several roles in viral infections, excellently depicted in [128]. An excellent review on how a viral infection can modulate mitochondrial functions has been recently published [3]. When focusing on the CNS, it becomes evident that strategies followed by sensu stricto neurotropic viruses can be shared with viruses infecting other cell types, while pandemic viruses, such as seasonal or zoonotic Influenza A viruses and SARS-CoV coronaviruses, can use functional receptors common to neuronal and non-neuronal cell types, such as, e.g., sialic acid or ACE-2, additionally interacting with proteins enriched in the nervous system, such as e.g., Neuropilin-1 [51,72].

Neurological disorders caused by infection with neurotropic viruses like Poliovirus, Herpes simplex, Rabies virus, Zika, and West-Nile viruses are often severe or even lethal, resulting in a wide spectrum of phenotypes including meningitis, encephalitis, paralysis, or newborn microcephaly [15,18,21,22,25,26,27], while neurological problems caused by Influenza A viruses [63,64] strongly vary, depending on the pathogenicity of the strain and co-morbidities [70,71].

More and more evidence is emerging about neurological complications in the wide sample of individuals infected worldwide because of the recent SARS-CoV-2 outbreak and ongoing pandemics. Indeed, the so-called COVID-19 has been mainly considered so far as a respiratory disease with immune system complications, while “Long COVID” clinical symptoms are often related to neurological disorders, including cognitive and behavioral impairment. Much research has focused so far on the interaction between the coronavirus Spike protein and ACE-2 as the main functional receptor, but a very important and possibly neglected role for Neuropilin-1 is emerging, and its connections with mitochondrial dynamics in determining neurological complications in COVID-19 and Long COVID open the route to further basic research, as well as to design of new therapeutic strategies [55,56,57]. 

In the context of CNS disorders, mitochondria bioenergetics and biogenesis might constitute a disease-biomarker to be assessed in therapies aimed at recovering neurological and behavioral impairments, through the reversion of the alteration in the gut microbiota. Mounting evidence suggests that the gut microbiota belongs to an intricate network that may concur to shape brain development and physiology. Nevertheless, more extensive studies are required to better characterize gut resident prokaryotes, both in healthy and pathological conditions. To that end, animal models of human brain pathologies constitute an invaluable system to perform omics-based longitudinal studies in controlled experimental conditions (i.e., with a genetic background, diet, and antibiotics treatment). Combinations of metagenomic (including epigenetics), metabolomic, and meta transcriptomic approaches might shed light on microbial composition and metabolites produced, and their molecular relationship with brain wellness. Effects on the GBA may be even ascribed to diet [129]. Accordingly, the identification of neuroactive molecules and whole signaling pathways involved within the GBA may help to better define the host-bacteria interaction, while exploring their translation outcomes.

From the therapeutic perspective, the gut-brain axis implies that microbiota can be targeted to improve neurological and neurodegenerative disorders and, vice versa, that treatments addressing neurological dysfunctions might impact the microbiota composition [130]. Current treatment protocols for both NDD and GI disorders may positively or adversely affect the composition of intestinal microbiota with a diverse impact on therapeutic outcome(s) [131]. Indeed, the gut microbiota can modify drug metabolism, and thereby impact drug efficacy and toxicity [132].

Genes responsible for neurodevelopmental disorders with multisystem phenotypes, even if mostly abundant in the brain, might fill relevant functions also in different body tissues. Nevertheless, the detrimental effects on CNS development and functions might mask the impact of inborn mutations outside the brain. While conditional null alleles enable the dissection of their function outside the CNS in mouse models, the investigations in humans can only rely on 3D cultures (organoids, organotypic, and bioprinted). In this regard, cancer-associated somatic mutations in NDD-associated genes can uncover specific tissue functions entailing systemic defects/comorbidities. As an example, the Glaass syndrome-associated gene SATB2 is selectively expressed in glandular cells of the lower gastrointestinal tract where it plays a vital role in maintaining intestinal homeostasis [133]. In intestinal epithelial-specific null mice with induced colitis, SATB2 deficiency promotes the disease development and colitis-associated colorectal cancer by influencing the intestinal luminal environment and gut flora (composition and infiltration) [134]. In mice and in human colonic organoids, SATB2 acts as a crucial determinant of mouse and human colonic stem cell fate [135]. Thus, studies of intestinal carcinoma, together with dietetic regimen and therapies of affected patients, may have beneficial readouts in CNS disorders with gut dysbiosis. Thus, from oncological research, we can gain new insight into pathological mechanisms and disease biomarkers for multisystemic disorders. Moreover, novel cancer-related therapeutics (e.g., Aptamer-linked small-interfering RNA Chimeras, Antisense Modified Oligonucleotides, or PROTAC) can also be reshaped for CNS disorders. Lastly, within the frame of precision medicine, the definition of personalized dietary programs would gain beneficial readouts in CNS disorders with gut dysbiosis and or metabolism unbalance. 

## Abbreviations

ACE-2	angiotensin converting enzyme 2
ARDS	acute respiratory distress syndrome
ASD	autism spectrum disorders
ATG5	autophagy related 5
BAD	Bcl-2 antagonist of cell death
BBB	brain blood barrier
BDNF	brain-derived neurotrophic factor
[Ca2+]	calcium concentration
CHPV	Chandipura Virus
CME	clathrin-mediated endocytosis
CNS	central nervous system
CoV	Coronavirus
COVID-19	coronavirus disease 2019
cyt	cytoplasmic
EC	endothelial cell
ER	endoplasmic reticulum
FTO	fat mass and obesity-associated gene
GBA	gut-brain axis
GBS	Guillain-Barre syndrome
GDFD	Growth retardation, developmental delay, coarse facies, and early death
GPCR	G-protein coupled receptor
HA	hemagglutinin
HAND	HIV-associated neurocognitive disorders
HCFC1	Host Cell Factor C1
HDAC	histone deacetylase
HIV	human immunodeficiency virus
HSE	HSV-1 encephalitis
HSV	Herpes simplex virus
HTLV-1	Human T-cell leukemia virus type 1
IFN-I	type I interferon
IP3R	inositol-1,4,5-triphosphate receptor
JEV	Japanese encephalitis virus
LDL	low-density lipoprotein
MAVS	mitochondria anti-viral signaling
MCU	mitochondrial calcium uniporter
MERS	Middle East Respiratory Syndrome
mt	mitochondrial
NA	neuraminidase
NDD	neurodevelopmental disorder
NMDAR	N-methyl-D-aspartate receptor
Nsp	non-structural protein
OMM	outer mitochondrial membrane
PI3K	phosphoinositide 3-kinase
PPA	propionate
PV	Poliovirus
RABV	Rabies virus
RIG-I	retinoic acid-inducible gene-I
ROS	reactive oxygen species
RSTS1	Rubinstein-Taybi syndromes
RTT	Rett syndrome
RyR	Ryanodine receptor
SARS	Severe Acute Respiratory Syndrome
SCFA	short-chain fatty acid
SIRT1	sirtuin 1
Tat	trans-activator of transcription
TMPRSS2	transmembrane serine protease 2
VDAC	voltage-dependent anion channel
VGCC	voltage gated calcium channel
WNV	West Nile virus

## References

[1] Mattson M.P., Haughey N.J., Nath A. (2005). Cell Death in HIV Dementia. Cell Death Differ..

[2] Gasparotto M., Lee Y.-S., Palazzi A., Vacca M., Filippini F. (2022). Nuclear and Cytoplasmatic Players in Mitochondria-Related CNS Disorders: Chromatin Modifications and Subcellular Trafficking. Biomolecules.

[3] Li X., Wu K., Zeng S., Zhao F., Fan J., Li Z., Yi L., Ding H., Zhao M., Fan S. (2021). Viral Infection Modulates Mitochondrial Function. Int. J. Mol. Sci..

[4] Buckley S., Byrnes S., Cochrane C., Roche M., Estes J.D., Selemidis S., Angelovich T.A., Churchill M.J. (2021). The Role of Oxidative Stress in HIV-Associated Neurocognitive Disorders. Brain Behav. Immun. Health.

[5] Himanshu D., Tandon R., Kumar S., Sawlani K., Verma S., Misra R., Atam V. (2022). Is International HIV Dementia Scale Good Enough to Diagnose HIV-Associated Neurocognitive Disorders?. J. Family Med. Prim. Care.

[6] Chaudhuri R., Arora H., Seth P. (2021). Mitochondrial Calcium Signaling in the Brain and Its Modulation by Neurotropic Viruses. Mitochondrion.

[7] Biasiotto R., Aguiari P., Rizzuto R., Pinton P., D’Agostino D.M., Ciminale V. (2010). The P13 Protein of Human T Cell Leukemia Virus Type 1 (HTLV-1) Modulates Mitochondrial Membrane Potential and Calcium Uptake. Biochim. Biophys. Acta (BBA)—Bioenerg..

[8] Ahmed H., Leyrolle Q., Koistinen V., Kärkkäinen O., Layé S., Delzenne N., Hanhineva K. (2022). Microbiota-Derived Metabolites as Drivers of Gut–Brain Communication. Gut. Microbes.

[9] Srikantha P., Mohajeri M.H. (2019). The Possible Role of the Microbiota-Gut-Brain-Axis in Autism Spectrum Disorder. Int. J. Mol. Sci..

[10] Dash S., Syed Y.A., Khan M.R. (2022). Understanding the Role of the Gut Microbiome in Brain Development and Its Association with Neurodevelopmental Psychiatric Disorders. Front. Cell Dev. Biol..

[11] Wikoff W.R., Anfora A.T., Liu J., Schultz P.G., Lesley S.A., Peters E.C., Siuzdak G. (2009). Metabolomics Analysis Reveals Large Effects of Gut Microflora on Mammalian Blood Metabolites. Proc. Natl. Acad. Sci. USA.

[12] Guo J.U., Su Y., Shin J.H., Shin J., Li H., Xie B., Zhong C., Hu S., Le T., Fan G. (2014). Distribution, Recognition and Regulation of Non-CpG Methylation in the Adult Mammalian Brain. Nat. Neurosci..

[13] Zhou Y., Frey T.K., Yang J.J. (2009). Viral Calciomics: Interplays between Ca^2+^ and Virus. Cell Calcium..

[14] Ghosh S., Mukherjee S., Sengupta N., Roy A., Dey D., Chakraborty S., Chattopadhyay D., Banerjee A., Basu A. (2016). Network Analysis Reveals Common Host Protein/s Modulating Pathogenesis of Neurotropic Viruses. Sci. Rep..

[15] Brisac C., Téoulé F., Autret A., Pelletier I., Colbère-Garapin F., Brenner C., Lemaire C., Blondel B. (2010). Calcium Flux between the Endoplasmic Reticulum and Mitochondrion Contributes to Poliovirus-Induced Apoptosis. J. Virol..

[16] Campanella M., de Jong A.S., Lanke K.W.H., Melchers W.J.G., Willems P.H.G.M., Pinton P., Rizzuto R., van Kuppeveld F.J.M. (2004). The Coxsackievirus 2B Protein Suppresses Apoptotic Host Cell Responses by Manipulating Intracellular Ca^2+^ Homeostasis. J. Biol. Chem..

[17] Zhang Q., Hsia S., Martin-Caraballo M. (2019). Regulation of T-type Ca^2+^ Channel Expression by Interleukin-6 in Sensory-like ND7/23 Cells Post-herpes Simplex Virus (HSV-1) Infection. J. Neurochem..

[18] Wnęk M., Ressel L., Ricci E., Rodriguez-Martinez C., Guerrero J.C.V., Ismail Z., Smith C., Kipar A., Sodeik B., Chinnery P.F. (2016). Herpes Simplex Encephalitis Is Linked with Selective Mitochondrial Damage; a Post-Mortem and in Vitro Study. Acta Neuropathol..

[19] Polansky H., Goral B. (2021). How an Increase in the Copy Number of HSV-1 during Latency Can Cause Alzheimer’s Disease: The Viral and Cellular Dynamics According to the Microcompetition Model. J. Neurovirol..

[20] Zan J., Liu J., Zhou J.-W., Wang H.-L., Mo K.-K., Yan Y., Xu Y.-B., Liao M., Su S., Hu R.-L. (2016). Rabies Virus Matrix Protein Induces Apoptosis by Targeting Mitochondria. Exp. Cell Res..

[21] Ubol S., Kasisith J., Pitidhammabhorn D., Tepsumethanol V. (2005). Screening of Pro-Apoptotic Genes Upregulated in an Experimental Street Rabies Virus-Infected Neonatal Mouse Brain. Microbiol. Immunol..

[22] Peng B.H., Wang T. (2019). West Nile Virus Induced Cell Death in the Central Nervous System. Pathogens.

[23] Kleinschmidt M.C., Michaelis M., Ogbomo H., Doerr H.-W., Cinatl J. (2007). Inhibition of Apoptosis Prevents West Nile Virus Induced Cell Death. BMC Microbiol..

[24] del Carmen Parquet M., Kumatori A., Hasebe F., Morita K., Igarashi A. (2001). West Nile Virus-Induced Bax-Dependent Apoptosis. FEBS Lett..

[25] Shrestha B., Gottlieb D., Diamond M.S. (2003). Infection and Injury of Neurons by West NileEncephalitisVirus. J. Virol..

[26] Samuel M.A., Morrey J.D., Diamond M.S. (2007). Caspase 3-Dependent Cell Death of Neurons Contributes to the Pathogenesis of West Nile Virus Encephalitis. J. Virol..

[27] Olmo I.G., Carvalho T.G., Costa V.V., Alves-Silva J., Ferrari C.Z., Izidoro-Toledo T.C., da Silva J.F., Teixeira A.L., Souza D.G., Marques J.T. (2017). Zika Virus Promotes Neuronal Cell Death in a Non-Cell Autonomous Manner by Triggering the Release of Neurotoxic Factors. Front. Immunol..

[28] Doñate-Macián P., Jungfleisch J., Pérez-Vilaró G., Rubio-Moscardo F., Perálvarez-Marín A., Diez J., Valverde M.A. (2018). The TRPV4 Channel Links Calcium Influx to DDX3X Activity and Viral Infectivity. Nat. Commun..

[29] Han X., Wang J., Yang Y., Qu S., Wan F., Zhang Z., Wang R., Li G., Cong H. (2021). Zika Virus Infection Induced Apoptosis by Modulating the Recruitment and Activation of Proapoptotic Protein Bax. J. Virol..

[30] Ledur P.F., Karmirian K., Pedrosa C.d.S.G., Souza L.R.Q., Assis-de-Lemos G., Martins T.M., Ferreira J.d.C.C.G., de Azevedo Reis G.F., Silva E.S., Silva D. (2020). Zika Virus Infection Leads to Mitochondrial Failure, Oxidative Stress and DNA Damage in Human IPSC-Derived Astrocytes. Sci. Rep..

[31] Heidari A., Righetto I., Filippini F. (2018). Electrostatic Variation of Haemagglutinin as a Hallmark of the Evolution of Avian Influenza Viruses. Sci. Rep..

[32] Lim Y., Ng Y., Tam J., Liu D. (2016). Human Coronaviruses: A Review of Virus–Host Interactions. Diseases.

[33] Cui J., Li F., Shi Z.-L. (2019). Origin and Evolution of Pathogenic Coronaviruses. Nat. Rev. Microbiol.

[34] Mostafa A., Kandeil A., Shehata M., el Shesheny R., Samy A.M., Kayali G., Ali M.A. (2020). Middle East Respiratory Syndrome Coronavirus (MERS-CoV): State of the Science. Microorganisms.

[35] Blanco-Melo D., Nilsson-Payant B.E., Liu W.-C., Uhl S., Hoagland D., Møller R., Jordan T.X., Oishi K., Panis M., Sachs D. (2020). Imbalanced Host Response to SARS-CoV-2 Drives Development of COVID-19. Cell.

[36] Kopecky-Bromberg S.A., Martínez-Sobrido L., Frieman M., Baric R.A., Palese P. (2007). Severe Acute Respiratory Syndrome Coronavirus Open Reading Frame (ORF) 3b, ORF 6, and Nucleocapsid Proteins Function as Interferon Antagonists. J. Virol..

[37] Shi C.-S., Qi H.-Y., Boularan C., Huang N.-N., Abu-Asab M., Shelhamer J.H., Kehrl J.H. (2014). SARS-Coronavirus Open Reading Frame-9b Suppresses Innate Immunity by Targeting Mitochondria and the MAVS/TRAF3/TRAF6 Signalosome. J. Immunol..

[38] Kreimendahl S., Rassow J. (2020). The Mitochondrial Outer Membrane Protein Tom70-Mediator in Protein Traffic, Membrane Contact Sites and Innate Immunity. Int. J. Mol. Sci..

[39] Fang P., Fang L., Zhang H., Xia S., Xiao S. (2021). Functions of Coronavirus Accessory Proteins: Overview of the State of the Art. Viruses.

[40] Padhan K., Minakshi R., Towheed M.A.B., Jameel S. (2008). Severe Acute Respiratory Syndrome Coronavirus 3a Protein Activates the Mitochondrial Death Pathway through P38 MAP Kinase Activation. J. Gen. Virol..

[41] Davies J.P., Almasy K.M., McDonald E.F., Plate L. (2020). Comparative Multiplexed Interactomics of SARS-CoV-2 and Homologous Coronavirus Nonstructural Proteins Identifies Unique and Shared Host-Cell Dependencies. ACS Infect. Dis..

[42] Zhang L., Wei L., Jiang D., Wang J., Cong X., Fei R. (2007). SARS-CoV Nucleocapsid Protein Induced Apoptosis of COS-1 Mediated by the Mitochondrial Pathway. Artif. Cells. Blood. Substit. Biotechnol..

[43] Chan C.-M., Ma C.-W., Chan W.-Y., Chan H.Y.E. (2007). The SARS-Coronavirus Membrane Protein Induces Apoptosis through Modulating the Akt Survival Pathway. Arch. Biochem. Biophys..

[44] Fu Y.-Z., Wang S.-Y., Zheng Z.-Q., Huang Y., Li W.-W., Xu Z.-S., Wang Y.-Y. (2021). SARS-CoV-2 Membrane Glycoprotein M Antagonizes the MAVS-Mediated Innate Antiviral Response. Cell Mol. Immunol..

[45] Wong S.H., Lui R.N., Sung J.J. (2020). Covid-19 and the Digestive System. J. Gastroenterol. Hepatol..

[46] Saleh J., Peyssonnaux C., Singh K.K., Edeas M. (2020). Mitochondria and Microbiota Dysfunction in COVID-19 Pathogenesis. Mitochondrion.

[47] Swain O., Romano S.K., Miryala R., Tsai J., Parikh V., Umanah G.K.E. (2021). SARS-CoV-2 Neuronal Invasion and Complications: Potential Mechanisms and Therapeutic Approaches. J. Neurosci..

[48] Kumari P., Rothan H.A., Natekar J.P., Stone S., Pathak H., Strate P.G., Arora K., Brinton M.A., Kumar M. (2021). Neuroinvasion and Encephalitis Following Intranasal Inoculation of SARS-CoV-2 in K18-HACE2 Mice. Viruses.

[49] Baig A.M., Khaleeq A., Ali U., Syeda H. (2020). Evidence of the COVID-19 Virus Targeting the CNS: Tissue Distribution, Host–Virus Interaction, and Proposed Neurotropic Mechanisms. ACS Chem. Neurosci..

[50] Rappoport J.Z., Benmerah A., Simon S.M. (2005). Analysis of the AP-2 Adaptor Complex and Cargo during Clathrin-Mediated Endocytosis. Traffic.

[51] Al-Thomali A.W., Al-kuraishy H.M., Al-Gareeb A.I., K. Al-buhadiliy A., de Waard M., Sabatier J.-M., Khan Khalil A.A., Saad H.M., Batiha G.E.-S. (2022). Role of Neuropilin 1 in COVID-19 Patients with Acute Ischemic Stroke. Biomedicines.

[52] Simonetti B., Daly J.L., Simón-Gracia L., Klein K., Weeratunga S., Antón-Plágaro C., Tobi A., Hodgson L., Lewis P.A., Heesom K.J. (2022). ESCPE-1 Mediates Retrograde Endosomal Sorting of the SARS-CoV-2 Host Factor Neuropilin-1. Proc. Natl. Acad. Sci. USA.

[53] Baindara P., Roy D., Mandal S.M., Schrum A.G. (2022). Conservation and Enhanced Binding of SARS-CoV-2 Omicron Spike Protein to Coreceptor Neuropilin-1 Predicted by Docking Analysis. Infect. Dis. Rep..

[54] Wang Y., Cao Y., Yamada S., Thirunavukkarasu M., Nin V., Joshi M., Rishi M.T., Bhattacharya S., Camacho-Pereira J., Sharma A.K. (2015). Cardiomyopathy and Worsened Ischemic Heart Failure in SM22-α Cre-Mediated Neuropilin-1 Null Mice. Arterioscler. Thromb. Vasc. Biol..

[55] Saleki K., Banazadeh M., Miri N.S., Azadmehr A. (2022). Triangle of Cytokine Storm, Central Nervous System Involvement, and Viral Infection in COVID-19: The Role of SFasL and Neuropilin-1. Rev. Neurosci..

[56] Issitt T., Bosseboeuf E., de Winter N., Dufton N., Gestri G., Senatore V., Chikh A., Randi A.M., Raimondi C. (2019). Neuropilin-1 Controls Endothelial Homeostasis by Regulating Mitochondrial Function and Iron-Dependent Oxidative Stress. iScience.

[57] Krasemann S., Haferkamp U., Pfefferle S., Woo M.S., Heinrich F., Schweizer M., Appelt-Menzel A., Cubukova A., Barenberg J., Leu J. (2022). The Blood-Brain Barrier Is Dysregulated in COVID-19 and Serves as a CNS Entry Route for SARS-CoV-2. Stem. Cell. Rep..

[58] Stefano G.B., Büttiker P., Weissenberger S., Martin A., Ptacek R., Kream R.M. (2021). Editorial: The Pathogenesis of Long-Term Neuropsychiatric COVID-19 and the Role of Microglia, Mitochondria, and Persistent Neuroinflammation: A Hypothesis. Med. Sci. Monit..

[59] Griffin D.E. (2010). Emergence and Re-Emergence of Viral Diseases of the Central Nervous System. Prog. Neurobiol..

[60] Shao W., Li X., Goraya M., Wang S., Chen J.-L. (2017). Evolution of Influenza A Virus by Mutation and Re-Assortment. Int. J. Mol. Sci..

[61] Righetto I., Milani A., Cattoli G., Filippini F. (2014). Comparative Structural Analysis of Haemagglutinin Proteins from Type A Influenza Viruses: Conserved and Variable Features. BMC Bioinform..

[62] de Bruin A.C.M., Funk M., Spronken M.I., Gultyaev A.P., Fouchier R.A.M., Richard M. (2022). Hemagglutinin Subtype Specificity and Mechanisms of Highly Pathogenic Avian Influenza Virus Genesis. Viruses.

[63] Lin X., Wang R., Zhang J., Sun X., Zou Z., Wang S., Jin M. (2015). Insights into Human Astrocyte Response to H5N1 Infection by Microarray Analysis. Viruses.

[64] Studahl M. (2003). Influenza Virus and CNS Manifestations. J. Clin. Virol.

[65] Siegers J.Y., van de Bildt M.W.G., Lin Z., Leijten L.M., Lavrijssen R.A.M., Bestebroer T., Spronken M.I.J., de Zeeuw C.I., Gao Z., Schrauwen E.J.A. (2019). Viral Factors Important for Efficient Replication of Influenza A Viruses in Cells of the Central Nervous System. J. Virol..

[66] Michalicová A., Bhide K., Bhide M., Kováč A. (2017). How Viruses Infiltrate the Central Nervous System. Acta. Virol..

[67] Davis C.O., Kim K.-Y., Bushong E.A., Mills E.A., Boassa D., Shih T., Kinebuchi M., Phan S., Zhou Y., Bihlmeyer N.A. (2014). Transcellular Degradation of Axonal Mitochondria. Proc. Natl. Acad. Sci. USA.

[68] Hayakawa K., Esposito E., Wang X., Terasaki Y., Liu Y., Xing C., Ji X., Lo E.H. (2016). Transfer of Mitochondria from Astrocytes to Neurons after Stroke. Nature.

[69] Ward M.C. (1996). Neurovirulence of Influenza A Virus. J. Neurovirol..

[70] Mori I., Yokochi T., Kimura Y. (2002). Role of Influenza A Virus Hemagglutinin in Neurovirulence for Mammalians. Med. Microbiol. Immunol..

[71] Hatta M., Gao P., Halfmann P., Kawaoka Y. (2001). Molecular Basis for High Virulence of Hong Kong H5N1 Influenza A Viruses. Science.

[72] Zhang X., Pu J., Sun Y., Bi Y., Jiang Z., Xu G., Zhang H., Cao J., Chang K.-C., Liu J. (2021). Neurovirulence of Avian Influenza Virus Is Dependent on the Interaction of Viral NP Protein with FMRP in the Murine Brain. J. Virol..

[73] Righetto I., Filippini F. (2020). Normal Modes Analysis and Surface Electrostatics of Haemagglutinin Proteins as Fingerprints for High Pathogenic Type A Influenza Viruses. BMC Bioinform..

[74] Zhang X., Xu G., Wang C., Jiang M., Gao W., Wang M., Sun H., Sun Y., Chang K.-C., Liu J. (2017). Enhanced Pathogenicity and Neurotropism of Mouse-Adapted H10N7 Influenza Virus Are Mediated by Novel PB2 and NA Mutations. J. Gen. Virol..

[75] Wang R., Zhu Y., Lin X., Ren C., Zhao J., Wang F., Gao X., Xiao R., Zhao L., Chen H. (2019). Influenza M2 Protein Regulates MAVS-Mediated Signaling Pathway through Interacting with MAVS and Increasing ROS Production. Autophagy.

[76] Pila-Castellanos I., Molino D., McKellar J., Lines L., da Graca J., Tauziet M., Chanteloup L., Mikaelian I., Meyniel-Schicklin L., Codogno P. (2021). Mitochondrial Morphodynamics Alteration Induced by Influenza Virus Infection as a New Antiviral Strategy. PLoS Pathog..

[77] Zeng Y., Xu S., Wei Y., Zhang X., Wang Q., Jia Y., Wang W., Han L., Chen Z., Wang Z. (2021). The PB1 Protein of Influenza A Virus Inhibits the Innate Immune Response by Targeting MAVS for NBR1-Mediated Selective Autophagic Degradation. PLoS Pathog..

[78] Long J.C.D., Fodor E. (2016). The PB2 Subunit of the Influenza A Virus RNA Polymerase Is Imported into the Mitochondrial Matrix. J. Virol..

[79] Liu J., Ji Q., Cheng F., Chen D., Geng T., Huang Y., Zhang J., He Y., Song T. (2022). The LncRNAs Involved in Regulating the RIG-I Signaling Pathway. Front. Cell Infect. Microbiol..

[80] Lin Y.-H., Platt M.P., Gilley R.P., Brown D., Dube P.H., Yu Y., Gonzalez-Juarbe N. (2021). Influenza Causes MLKL-Driven Cardiac Proteome Remodeling during Convalescence. Circ. Res..

[81] Tran A.T., Cortens J.P., Du Q., Wilkins J.A., Coombs K.M. (2013). Influenza Virus Induces Apoptosis via BAD-Mediated Mitochondrial Dysregulation. J. Virol..

[82] Bian Q., Lu J., Zhang L., Chi Y., Li Y., Guo H. (2017). Highly Pathogenic Avian Influenza A Virus H5N1 Non-structural Protein 1 Is Associated with Apoptotic Activation of the Intrinsic Mitochondrial Pathway. Exp. Ther. Med..

[83] Zhu Y., Li Y., Zhang Q., Song Y., Wang L., Zhu Z. (2022). Interactions Between Intestinal Microbiota and Neural Mitochondria: A New Perspective on Communicating Pathway from Gut to Brain. Front. Microbiol..

[84] Blaser M.J. (2014). The Microbiome Revolution. J. Clin. Invest..

[85] Nicholson J.K., Holmes E., Kinross J., Burcelin R., Gibson G., Jia W., Pettersson S. (2012). Host-Gut Microbiota Metabolic Interactions. Science.

[86] Fung T.C., Olson C.A., Hsiao E.Y. (2017). Interactions between the Microbiota, Immune and Nervous Systems in Health and Disease. Nat. Neurosci..

[87] Midtvedt A.-C., Midtvedt T. (1992). Production of Short Chain Fatty Acids by the Intestinal Microflora during the First 2 Years of Human Life. J. Pediatr. Gastroenterol. Nutr..

[88] Pistollato F., Sumalla Cano S., Elio I., Masias Vergara M., Giampieri F., Battino M. (2016). Role of Gut Microbiota and Nutrients in Amyloid Formation and Pathogenesis of Alzheimer Disease. Nutr. Rev..

[89] Mulak A. (2015). Brain-Gut-Microbiota Axis in Parkinson’s Disease. World J. Gastroenterol..

[90] Cristiano C., Lama A., Lembo F., Mollica M.P., Calignano A., Mattace Raso G. (2018). Interplay Between Peripheral and Central Inflammation in Autism Spectrum Disorders: Possible Nutritional and Therapeutic Strategies. Front. Physiol..

[91] Neier K., Grant T.E., Palmer R.L., Chappell D., Hakam S.M., Yasui K.M., Rolston M., Settles M.L., Hunter S.S., Madany A. (2021). Sex Disparate Gut Microbiome and Metabolome Perturbations Precede Disease Progression in a Mouse Model of Rett Syndrome. Commun. Biol..

[92] Arnold J.W., Roach J., Azcarate-Peril M.A. (2016). Emerging Technologies for Gut Microbiome Research. Trends Microbiol..

[93] den Besten G., van Eunen K., Groen A.K., Venema K., Reijngoud D.-J., Bakker B.M. (2013). The Role of Short-Chain Fatty Acids in the Interplay between Diet, Gut Microbiota, and Host Energy Metabolism. J. Lipid. Res..

[94] Kaur H., Singh Y., Singh S., Singh R.B. (2021). Gut Microbiome-Mediated Epigenetic Regulation of Brain Disorder and Application of Machine Learning for Multi-Omics Data Analysis. Genome.

[95] Hylemon P.B., Zhou H., Pandak W.M., Ren S., Gil G., Dent P. (2009). Bile Acids as Regulatory Molecules. J. Lipid. Res..

[96] Joyce S.A., Gahan C.G.M. (2016). Bile Acid Modifications at the Microbe-Host Interface: Potential for Nutraceutical and Pharmaceutical Interventions in Host Health. Annu. Rev. Food Sci. Technol..

[97] Sugahara H., Odamaki T., Hashikura N., Abe F., Xiao J. (2015). Differences in Folate Production by Bifidobacteria of Different Origins. Biosci. Microbiota Food Health.

[98] Marley M.G., Meganathan R., Bentley R. (1986). Menaquinone (Vitamin K2) Biosynthesis in Escherichia Coli: Synthesis of o-Succinylbenzoate Does Not Require the Decarboxylase Activity of the Ketoglutarate Dehydrogenase Complex. Biochemistry.

[99] Mao J.-H., Kim Y.-M., Zhou Y.-X., Hu D., Zhong C., Chang H., Brislawn C.J., Fansler S., Langley S., Wang Y. (2020). Genetic and Metabolic Links between the Murine Microbiome and Memory. Microbiome.

[100] Cani P.D., Knauf C. (2016). How Gut Microbes Talk to Organs: The Role of Endocrine and Nervous Routes. Mol. Metab..

[101] Luu M., Visekruna A. (2019). Short-chain Fatty Acids: Bacterial Messengers Modulating the Immunometabolism of T Cells. Eur. J. Immunol..

[102] Melbye P., Olsson A., Hansen T.H., Søndergaard H.B., Bang Oturai A. (2019). Short-Chain Fatty Acids and Gut Microbiota in Multiple Sclerosis. Acta Neurol. Scand..

[103] Chen J., Vitetta L. (2020). Butyrate in Inflammatory Bowel Disease Therapy. Gastroenterology.

[104] Rose S., Niyazov D.M., Rossignol D.A., Goldenthal M., Kahler S.G., Frye R.E. (2018). Clinical and Molecular Characteristics of Mitochondrial Dysfunction in Autism Spectrum Disorder. Mol. Diagn. Ther..

[105] Uittenbogaard M., Brantner C.A., Chiaramello A. (2018). Epigenetic Modifiers Promote Mitochondrial Biogenesis and Oxidative Metabolism Leading to Enhanced Differentiation of Neuroprogenitor Cells. Cell Death Dis..

[106] Blevins H.M., Xu Y., Biby S., Zhang S. (2022). The NLRP3 Inflammasome Pathway: A Review of Mechanisms and Inhibitors for the Treatment of Inflammatory Diseases. Front Aging Neurosci..

[107] Yu Y., Wang H., Rao X., Liu L., Zheng P., Li W., Zhou W., Chai T., Ji P., Song J. (2021). Proteomic Profiling of Lysine Acetylation Indicates Mitochondrial Dysfunction in the Hippocampus of Gut Microbiota-Absent Mice. Front Mol. Neurosci..

[108] el Hayek L., Khalifeh M., Zibara V., Abi Assaad R., Emmanuel N., Karnib N., El-Ghandour R., Nasrallah P., Bilen M., Ibrahim P. (2019). Lactate Mediates the Effects of Exercise on Learning and Memory through SIRT1-Dependent Activation of Hippocampal Brain-Derived Neurotrophic Factor (BDNF). J. Neurosci..

[109] Cohen H.Y., Miller C., Bitterman K.J., Wall N.R., Hekking B., Kessler B., Howitz K.T., Gorospe M., de Cabo R., Sinclair D.A. (2004). Calorie Restriction Promotes Mammalian Cell Survival by Inducing the SIRT1 Deacetylase. Science.

[110] Pan W.-H., Sommer F., Falk-Paulsen M., Ulas T., Best P., Fazio A., Kachroo P., Luzius A., Jentzsch M., Rehman A. (2018). Exposure to the Gut Microbiota Drives Distinct Methylome and Transcriptome Changes in Intestinal Epithelial Cells during Postnatal Development. Genome. Med..

[111] Vaquero A., Scher M., Lee D., Erdjument-Bromage H., Tempst P., Reinberg D. (2004). Human SirT1 Interacts with Histone H1 and Promotes Formation of Facultative Heterochromatin. Mol. Cell.

[112] Adams J.B., Johansen L.J., Powell L.D., Quig D., Rubin R.A. (2011). Gastrointestinal Flora and Gastrointestinal Status in Children with Autism—Comparisons to Typical Children and Correlation with Autism Severity. BMC Gastroenterol..

[113] Motil K.J., Caeg E., Barrish J.O., Geerts S., Lane J.B., Percy A.K., Annese F., McNair L., Skinner S.A., Lee H.-S. (2012). Gastrointestinal and Nutritional Problems Occur Frequently throughout Life in Girls and Women with Rett Syndrome. J. Pediatr. Gastroenterol. Nutr..

[114] Milani D., Manzoni F.M.P., Pezzani L., Ajmone P., Gervasini C., Menni F., Esposito S. (2015). Rubinstein-Taybi Syndrome: Clinical Features, Genetic Basis, Diagnosis, and Management. Ital. J. Pediatr..

[115] Rossignol D.A., Frye R.E. (2012). Mitochondrial Dysfunction in Autism Spectrum Disorders: A Systematic Review and Meta-Analysis. Mol. Psychiatry.

[116] MacFabe D.F. (2012). Short-Chain Fatty Acid Fermentation Products of the Gut Microbiome: Implications in Autism Spectrum Disorders. Microb. Ecol. Health Dis..

[117] Jones L.L., McDonald D.A., Borum P.R. (2010). Acylcarnitines: Role in Brain. Prog. Lipid. Res..

[118] Justice M.J., Buchovecky C.M., Kyle S.M., Djukic A. (2013). A Role for Metabolism in Rett Syndrome Pathogenesis. Rare Dis..

[119] Derecki N.C., Cronk J.C., Kipnis J. (2015). Microglia Involvement in Rett Syndrome. Neuroinflammation.

[120] Borghi E., Borgo F., Severgnini M., Savini M., Casiraghi M., Vignoli A. (2017). Rett Syndrome: A Focus on Gut Microbiota. Int. J. Mol. Sci..

[121] Strati F., Cavalieri D., Albanese D., de Felice C., Donati C., Hayek J., Jousson O., Leoncini S., Pindo M., Renzi D. (2016). Altered Gut Microbiota in Rett Syndrome. Microbiome.

[122] di Fede E., Ottaviano E., Grazioli P., Ceccarani C., Galeone A., Parodi C., Colombo E.A., Bassanini G., Fazio G., Severgnini M. (2021). Insights into the Role of the Microbiota and of Short-Chain Fatty Acids in Rubinstein–Taybi Syndrome. Int. J. Mol. Sci..

[123] Daoud H., Zhang D., McMurray F., Yu A., Luco S.M., Vanstone J., Jarinova O., Carson N., Wickens J., Shishodia S. (2016). Identification of a Pathogenic *FTO* Mutation by next-Generation Sequencing in a Newborn with Growth Retardation and Developmental Delay. J. Med. Genet..

[124] Mauer J., Luo X., Blanjoie A., Jiao X., Grozhik A.v., Patil D.P., Linder B., Pickering B.F., Vasseur J.-J., Chen Q. (2017). Reversible Methylation of M6Am in the 5′ Cap Controls MRNA Stability. Nature.

[125] Sun L., Ma L., Zhang H., Cao Y., Wang C., Hou N., Huang N., von Deneen K.M., Zhao C., Shi Y. (2019). *Fto* Deficiency Reduces Anxiety- and Depression-Like Behaviors in Mice via Alterations in Gut Microbiota. Theranostics.

[126] Yu H.-C., Sloan J.L., Scharer G., Brebner A., Quintana A.M., Achilly N.P., Manoli I., Coughlin C.R., Geiger E.A., Schneck U. (2013). An X-Linked Cobalamin Disorder Caused by Mutations in Transcriptional Coregulator HCFC1. Am. J. Hum. Genet..

[127] Esser A.J., Mukherjee S., Dereven’kov I.A., Makarov S.v., Jacobsen D.W., Spiekerkoetter U., Hannibal L. (2022). Versatile Enzymology and Heterogeneous Phenotypes in Cobalamin Complementation Type C Disease. iScience.

[128] Elesela S., Lukacs N.W. (2021). Role of Mitochondria in Viral Infections. Life.

[129] Obrenovich M. (2018). Leaky Gut, Leaky Brain?. Microorganisms.

[130] Maier L., Pruteanu M., Kuhn M., Zeller G., Telzerow A., Anderson E.E., Brochado A.R., Fernandez K.C., Dose H., Mori H. (2018). Extensive Impact of Non-Antibiotic Drugs on Human Gut Bacteria. Nature.

[131] Holmes M., Flaminio Z., Vardhan M., Xu F., Li X., Devinsky O., Saxena D. (2020). Cross Talk between Drug-resistant Epilepsy and the Gut Microbiome. Epilepsia.

[132] Kitamura S., Sugihara K., Kuwasako M., Tatsumi K. (2011). The Role of Mammalian Intestinal Bacteria in the Reductive Metabolism of Zonisamide. J. Pharm. Pharmacol..

[133] Li Z., Yuan J., Wei L., Zhou L., Mei K., Yue J., Gao H., Zhang M., Jia L., Kang Q. (2015). SATB2 Is a Sensitive Marker for Lower Gastrointestinal Well-Differentiated Neuroendocrine Tumors. Int. J. Clin. Exp. Pathol..

[134] Ni H., Chen Y., Xia W., Wang C., Hu C., Sun L., Tang W., Cui H., Shen T., Liu Y. (2021). SATB2 Defect Promotes Colitis and Colitis-Associated Colorectal Cancer by Impairing Cl^−^/HCO^3−^ Exchange and Homeostasis of Gut Microbiota. J. Crohns. Colitis.

[135] Gu W., Wang H., Huang X., Kraiczy J., Singh P.N.P., Ng C., Dagdeviren S., Houghton S., Pellon-Cardenas O., Lan Y. (2022). SATB2 Preserves Colon Stem Cell Identity and Mediates Ileum-Colon Conversion via Enhancer Remodeling. Cell. Stem. Cell.

